# Medium-Long-Term Clinical and Radiographic Outcomes of Minimally Invasive Distal Metatarsal Metaphyseal Osteotomy (DMMO) for Central Primary Metatarsalgia: Do Maestro Criteria Have a Predictive Value in the Preoperative Planning for This Percutaneous Technique?

**DOI:** 10.1155/2018/1947024

**Published:** 2018-11-15

**Authors:** Carlo Biz, Marco Corradin, Wilfried Trepin Kuete Kanah, Miki Dalmau-Pastor, Alessandro Zornetta, Andrea Volpin, Pietro Ruggieri

**Affiliations:** ^1^Orthopaedic Clinic, Department of Surgery, Oncology and Gastroenterology DiSCOG, University of Padua, Via Giustiniani 2, Padova, Italy; ^2^Human Anatomy and Embryology Unit, Experimental Pathology and Therapeutics Department, University of Barcelona, Barcelona, Spain; ^3^Health Sciences Faculty of Manresa, University of Vic-Central University of Catalunya, Vic, Spain; ^4^GRECMIP: Groupe de Recherche et d'Etude en Chirurgie Mini-Invasive du Pied, Merignac, France; ^5^Department of Trauma & Orthopaedic Surgery, Royal Derby Hospital NHS Foundation Trust, Uttoxeter Road, Derby, UK

## Abstract

**Background:**

The purpose of this prospective study was first to evaluate the safety and effectiveness of Minimally Invasive Distal Metatarsal Metaphyseal Osteotomy (DMMO) in treating central metatarsalgia, identifying possible contraindications. The second objective was to verify the potential of DMMO to restore a harmonious forefoot morphotype according to Maestro criteria.

**Methods:**

A consecutive series of patients with metatarsalgia was consecutively enrolled and treated by DMMO. According to Maestro criteria, preoperative planning was carried out by both clinical and radiological assessment. Patient demographic data, AOFAS scores, 17-FFI, MOXFQ, SF-36, VAS, and complications were recorded. Maestro parameters, relative morphotypes, and bone callus formation were assessed. Statistical analysis was carried out (*p *< 0.05).

**Results:**

Ninety-three patients (93 feet) with a mean age of 62.4 (31-87) years were evaluated. At mean follow-up of 58.7 (36-96) months, all of the clinical scores improved significantly (*p* < 0.0001). Most of the osteotomies (76.3%) had healed by 3-month follow-up, while ideal harmonious morphotype was restored only in a few feet (3.2%). Clinical and radiological outcomes were not different based on principal demographic parameters. Long-term complications were recorded in 12 cases (12.9%).

**Conclusion:**

DMMO is a safe and effective method for the treatment of metatarsalgia. Although Maestro criteria were useful to calculate the metatarsal bones to be shortened and a significant clinical improvement of all scores was achieved, the ideal harmonious morphotype was restored only in a few feet. Hence, our data show that Maestro criteria did not have a predictive value in clinical outcomes of DMMO.

## 1. Introduction

Central metatarsalgia, one of the most common problems in orthopaedic clinical practice, is a term used to indicate a painful condition localized in the plantar forefoot region between the 2nd and 4th metatarsal heads and/or the metatarsophalangeal joint [1–3], often associated with the most frequent forefoot disorders, such as hallux valgus (HV) and lesser toe deformities. Ten percent of the general population has had some form of pain in the metatarsal region during their lifetime, the majority being female [4, 5]. This condition is not a diagnosis but a symptom for which there can be several contributing factors [6]. Metatarsalgia syndrome can be classified as primary or biomechanical, secondary, and iatrogenic [3, 7, 8]. Primary or biomechanical metatarsalgia is the most important, accounting for about 90% of cases [2]. It is caused by intrinsic abnormalities of metatarsal anatomy and the relationship between the metatarsal bones (MBs) and the rest of the foot, resulting in an overload to the forefoot [5]. Its most common cause is a long second MB [9]. Individuals presenting congenital brevity of the 1st metatarsal (M1) or severe HV may complain of pain in the forefoot caused by incompetence of the first ray in its weight-bearing function, creating transfer pressure to lesser metatarsal, in particular the 2nd metatarsal (M2). Other causes of primary metatarsalgia include disproportionate length of M2 or the 3rd metatarsal (M3), congenital deformities of the metatarsal heads, tightness of the gastrocnemius muscles or triceps, fixed equinus of the foot, pes cavus, and any hindfoot abnormality that results in overloading of the forefoot [10, 11]. Maestro and Besse have studied forefoot structure to define ideal forefoot morphology with precise criteria [9]. Forefoot alterations cause imbalance in weight-bearing distribution that may lead to mechanical overload on the affected metatarsal heads and may evolve to pain and plantar callosities [4, 12].

First-step metatarsalgia treatment is conservative, including physical therapy and stretching exercises for gastrocsoleous tightness, functional foot orthoses and shoe modifications to lessen the pressure on the forefoot, debridement of calluses associated with painful keratosis when present, and judicious use of corticosteroid injections [13]. These can be sufficient to achieve satisfactory results in 85% of cases [8] and should always be carried out before considering surgery in the management of this condition [3].

However, when these conservative measures fail and the metatarsalgia becomes recalcitrant, it requires surgical treatment with or without procedures on the first ray [14, 15]. The primary goal of surgery is to relieve pain and restore an ideal forefoot morphology with a normal distribution of pressure in the forefoot [16–18]. Multiple surgical procedures have been described for this syndrome [1, 19–26]. However, the Weil osteotomy (and its modifications) has been the preferred technique employed by surgeons for many years in Europe [17, 26, 27]. This procedure consists of an open intra-articular osteotomy performed after preoperative planning based on the Maestro's criteria to calculate the appropriate metatarsal length from anteroposterior standing radiographs [9, 11, 27, 28], which provides longitudinal decompression. Although it was originally designed to restore the physiologic cascade of the lesser MBs and evenly redistribute the pressures on the forefoot, the main complication, estimated to be among at least 10 to 30% of cases, is postoperative stiffness, while other commonly described problems include floating toe, recurrence, and transfer metatarsalgia [8, 29–32]. For these reasons, this procedure has generated considerable controversy.

Within the last decade, innovative Distal Metatarsal Metaphyseal Osteotomy (DMMO) has proved to be an alternative surgical approach, being a percutaneous extra-articular metatarsal neck osteotomy without any internal fixation [19, 20, 29], which permits the metatarsal lengths to be set automatically upon weight bearing. This technique results in less postoperative stiffness than the standard Weil osteotomy [17]. More recently, this percutaneous procedure was modified to reduce the plantar pressure of the MBs over chronic plantar ulcers in diabetic patients, promoting ulcer healing [33].

Hence, the primary purpose of this prospective study was to specifically evaluate the safety and effectiveness of Minimally Invasive Distal Metatarsal Metaphyseal Osteotomy (DMMO) in treating patients with persistent central primary metatarsalgia, associated or not with HV and lesser toe deformities, identifying possible contraindications in relation to some demographic parameters (age, gender, BMI, and smoking). The second objective was to verify the potential of DMMO in restoring a harmonious foot morphotype according to Maestro's criteria and if these radiographic parameters are correlated with clinical outcomes, maintaining the predictive value of these criteria during preoperative planning also for this percutaneous surgery.

## 2. Material and Methods

### 2.1. Patients

At our institution, between January 2009 and December 2013, a consecutive series of 131 Caucasian patients with diagnosis of central primary metatarsalgia resistant to conservative treatment was enrolled in this prospective study. All patients underwent DMMO, performed by a single surgeon, the senior author (C.B.), trained in minimally invasive surgery (MIS), who followed and checked the patients personally during the postoperative period. All subjects participating in this study received a thorough explanation of the risks and benefits of inclusion and gave their oral and written informed consent to publish the data. This study was approved by the Institutional Ethics Committee and performed in accordance with the ethical standards of the 1964 Declaration of Helsinki as revised in 2000 and those of Good Clinical Practice.

### 2.2. Inclusion and Exclusion Criteria

Patients with diagnosis of central primary metatarsalgia between M2 and M4 of a biomechanical etiology in the plantar foot area were enrolled consecutively and prospectively with precise inclusion criteria over a 5-year period. Ages ranged from 18 to 90 years. Only symptomatic patients with persistent pain, with or without forefoot plantar hyperkeratosis lesions, unresponsive to conservative and orthotic treatment performed for at least 6 months, were included in this study and underwent DMMO on a single foot. Associated forefoot pathologies included HV, metatarsophalangeal (MTP) joint instability or dislocation, and flexible or fixed lesser toe deformities. Exclusion criteria were as follows: arthritis and stiffness of MTP joint, congenital deformities of the foot, hallux rigidus, Freiberg's infraction, Morton's neuroma, and diagnosis of rheumatic, metabolic, neurologic, infective, or psychiatric pathologies. Furthermore, patients were excluded if they had previous trauma or foot and ankle surgery, or any form of secondary or iatrogenic metatarsalgia.

### 2.3. Preoperative Planning

**Figure 1:**
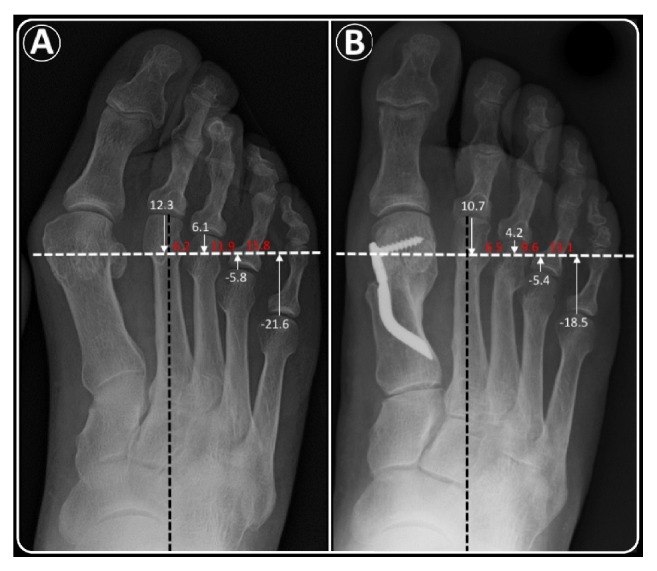
*Maestro criteria*: example of radiographic marks, relative measurements (millimeters in white) for each lesser MB, and their difference with the consecutive one (millimeters in red) on anteroposterior radiographs of a forefoot of our series, performed to calculate the number of MBs to be shortened during radiographic planning and to identify the corresponding morphotype pre- and postoperatively. According to these criteria, the forefoot was classified as* “unclassified non-harmonious”* in the preoperative period (A) and as* “non-harmonious 1”* at the last follow-up after DMMO (B). Endolog technique was also performed for HV correction.

Both clinical and radiological assessment were used for preoperative planning. The general aspects of metatarsalgia were evaluated: affected side, plantar hyperkeratosis lesions, site of metatarsalgia, symptomatic MTP joint instability (using the Lachman test), and relative clinical signs of dorsal dislocation. With these data, it was decided where the osteotomy should lead to rebalance plantar pressures and create a harmonious curve, with a tolerance of ±1 mm for Maestro criteria 1 and 2, ±2 mm for Maestro criteria 3. A normal or harmonious forefoot shows a geometrical progression of 2 regarding the relative lengths of the lesser metatarsals compared to the SM4 line, the line passing through the mid-third of the M4 head (+2 mm proximally/center M4 head/-4 mm distally). The line of the osteotomy was drawn from the center of the lateral sesamoid through the central or distal third of the M4 head, perpendicular to the axis of the foot (Figure 1). The DMMO was carried out only on the metatarsal of symptomatic MTP joints unless this shortening would make the neighboring metatarsal too long, resulting in a disharmonious morphotype with a high risk of a transfer lesion. The adjacent metatarsal was also shortened in these cases.

In a second step, associated deformities, when present, were assessed and then corrected during the same operation. Surgical procedures on the first ray were performed according to our institutional protocol: HV correction by Reverdin-Isham percutaneous osteotomy for mild-moderate deformity, or Endolog technique for moderate-severe deformity, both generally followed by percutaneous Akin osteotomy [29, 34]. In addition, percutaneous lateral soft-tissue release and percutaneous tenotomy of extensor and/or flexor, in association with (or not) phalange percutaneous osteotomies, were tailored based on the lesser toe deformities, flexible or fixed.

### 2.4. Operative Technique

**Figure 2:**
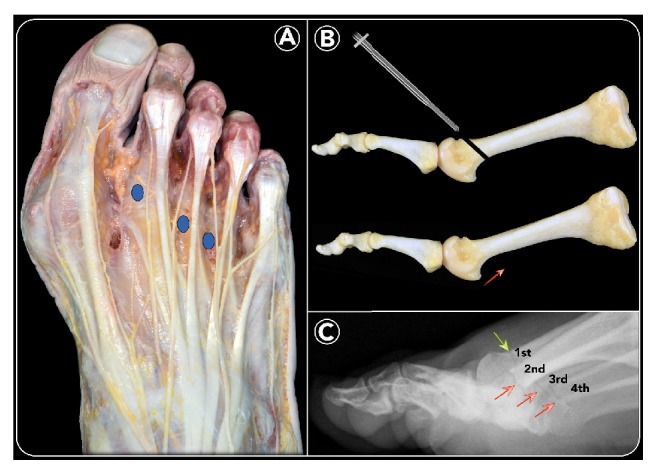
*Distal Metatarsal Metaphyseal Osteotomy (DMMO): *dissection of the dorsum of the foot showing the extensor apparatus and the innervation pattern of the foot and toes. Blue circles highlight the incision points for percutaneous DMMO (A). Bone preparation of an ideal DMMO procedure performed at the level of the M-neck and the final result showing the shortening and elevation of the M-head (B). Weight-bearing lateral radiographic view showing the site of the DMMO (red arrow) on each lesser MB; green arrow corresponds to Reverdin-Isham osteotomy for HV correction during the same operation (C).

The surgical technique is performed according to the same principles and the same general indications described by M. De Prado [20, 21] (Figure 2). During the operation, the patient is in a supine position, with the operated foot protruding from the table. No ankle joint tourniquet is applied, as it is not required for this technique. Prophylactic antibiotic (Cefazolin: 2 g) is administered before surgery. The anesthesiologist performs a regional block of the foot, involving superficial nerves (saphenous, sural, and superficial peroneal) and deep nerves (deep peroneal and tibial).

### 2.5. Minimally Invasive Distal Metatarsal Metaphyseal Osteotomy (DMMO)

**Figure 3:**
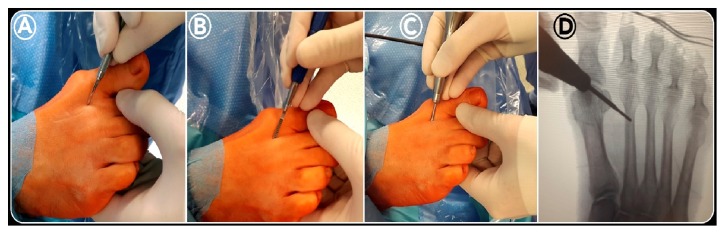
*DMMO intraoperative images (1st):* using a small scalpel blade (SM64), an incision of 3-5 mm was made parallel to the extensor tendons at the dorsal side of the medial border of each M-head that needed to be shortened. The scalpel was advanced at an oblique angle of about 45° until it reached the dorsal aspect of the distal MB at the level of the neck (A). Through the same incision, first a bone rasp was inserted, using it to separate the periosteum at the level of osteotomy (B). Then, a Shannon Isham burr (2.0 × 12 mm) was introduced until it reached the metatarsal neck (C). Fluoroscopy was used to confirm the correct position of the osteotomy site on the distal metaphysis of the MB (D).

**Figure 4:**
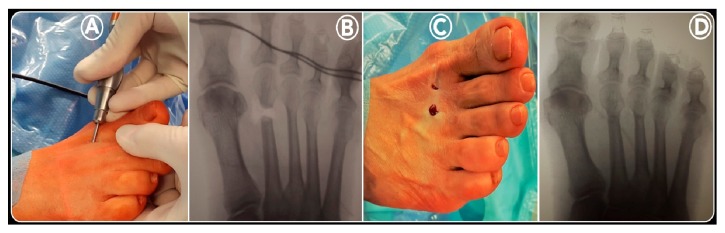
*DMMO intraoperative images (2nd):* in this position, the DMMO was performed with an angle of approximately 45° with respect to the long axis of the MB in a dorsal-distal to proximal-plantar direction, with rotary motion, extending to the contralateral cortex (A). To verify the completion of the osteotomy of each MB operated on, manual traction on the corresponding toe was applied under fluoroscopic control (B). Before closing the wounds by resorbable sutures (C), the MBs were manually compacted, applying pressure in the distal-proximal direction, pushing their heads dorsally. Finally, after bandage application, a final radiographic check was made to evaluate the correction obtained (D).

The surgeon holds the metatarsophalangeal joint between the thumb and index finger of his nondominant hand [19]. Using a small scalpel blade (SM64) in his dominant hand, an incision of 5 mm is made parallel to the extensor tendons at the dorsal side of the medial (or lateral) border of each metatarsal head that needs to be shortened (Figures 2(A) and 3(A)) [14]. The side of the incision depends on the surgeon's handedness and which foot is being operated on. The scalpel is advanced at an oblique angle of about 45° until it reaches the dorsal aspect of the distal MB, at the neck level, to undergo osteotomy (Figure 3(A)). Through the same incision, first a bone rasp specific for percutaneous surgery is inserted, using it to separate the periosteum at the level of the osteotomy site (Figure 3(B)). Then, a Shannon Isham burr (2.0 × 12 mm), adapted for Mm960 (produced by Medic Micro, Switzerland), is introduced until it reaches the metatarsal neck where the periosteum was previously removed (Figure 3(C)). Fluoroscopy is used to confirm the correct position of the osteotomy site on the distal diaphysis of the MB (Figure 3(D)). In this position, the cutting is started with an angle of approximately 45° with respect to the long axis of the MB in a dorsal-distal to proximal-plantar direction, with rotary motion, extending to the contralateral cortex (Figures 2(B) and 4(A)). In this way, the lateral cortical surface is cut first, then the plantar, medial, and, lastly, the dorsal cortical surface. During the osteotomy process, the incision site is irrigated by normal saline, as the burr can cause excessive heat, causing first skin burn and resulting subsequently in fibrosis and pseudoarthrosis at the bone level. Further, this lavage is useful to remove bone debris, preventing periarticular ossifications in the stab canal. To verify the completion of the osteotomy of each MB operated on, manual traction on the corresponding toe was applied under fluoroscopic control (Figure 4(B)). Upon completion of the osteotomy, the bone is manually compacted, applying pressure in the distal-proximal direction of the interested metatarsal, pushing the metatarsal head dorsally and producing contact of the trabecular bone, since no internal fixation is performed (Figure 2(C)). In our cohort, the number of metatarsal osteotomies performed in each forefoot was planned according to how much the metatarsal formula was altered according to the Maestro criteria (Figure 1). Before closing the wounds by resorbable sutures (Figure 4(C)), a final radiographic check was made to evaluate the correction obtained (Figure 2(D)).

### 2.6. Bandage

Because there is no osteosynthesis material in this surgery, the bandage is a very important tool in order to maintain the metatarsal head position achieved with the operation. Consequently, its application was performed with the utmost care and attention. The crisscross bandage was traced between all intermetatarsal spaces, crossing it over the medial (lateral) aspect of all of the osteotomies performed in order to reinforce the strength of the bandage. Gentle traction was used to maintain the toe in light hypercorrection and plantar inclination. Finally, the forefoot was covered with tubular gauzes, except for the distal part of the toes and nails.

### 2.7. Postoperative Protocol

All patients followed the same postoperative protocol and were followed in the same standardized manner by the senior author (C.B.). The patients were allowed to walk as much as they could tolerate the day after surgery using a rigid flat-soled orthopedic shoe for the following 30-day period. The patient was discharged the same day, warned of the possible persistence of swelling for 1-3 months, of pain, and of the presence of clicks in the forefoot due to movements in the osteotomy sites. This factor is very important as metatarsal length sets automatically upon weight bearing of the foot [9, 14]. Anteroposterior and lateral X-rays of non-weight-bearing feet were taken before the patients were discharged. We recommended an antibiotic oral prophylaxis for a week, as well as thromboembolic prophylaxis (Natrium Enoxaparin: 4.000 IU/day) and an anti-edemigen therapy (Leucoselect, Lymphaselect, and Bromelina: 1 tablet/day) for 30 days starting from the day of the surgery. Moreover, an analgesic therapy was prescribed for 2 weeks of Etoricoxib (90 or 60 mg, 1 tablet/day) in the morning, also to prevent heterotopic ossification when the comorbidities of the patient permitted, or, alternatively, Paracetamol (1g, 1 tablet 2x/day). All of the patients were seen once a week for a month in our outpatient clinic. The first visit was 8 days after surgery. The original bandage was removed and substituted by a simpler bandage. During the 3 weekly visits, the bandage was changed in the same way. One month after surgical treatment, the bandage was totally removed after taking anteroposterior weight-bearing and lateral X-rays. The patients were then able to walk with comfortable shoes, allowing total load on the operated foot. No specific physiokinetic therapy was suggested to restart daily activities. However, controlled return to sports was not allowed for 3 months after surgery.

### 2.8. Patient Assessment

The clinical and radiological analyses were carried out, respectively, by two independent investigators, the junior authors (W.T.K.K. and A.Z.), not involved in the primary surgical treatment of the patients. For this study, all patients were subjected to clinical and radiographic evaluation with the same protocol prior to surgery as well as regular follow-ups, following our institutional standard aftercare algorithm, according to this study protocol, to AOFAS accepted guidelines [15, 35], and based on the Maestro criteria [9]. For methodological reasons, the immediate postoperative X-rays at discharge, as well as the one-month radiographic control, were not included for the radiographic evaluation: the first because it was a non-weight-bearing radiograph; the second because although it was prescribed as weight-bearing, in some cases it was not performed because the patients had pain or were afraid to place the operated foot on the ground without an orthopedic shoe. Finally, the clinical-functional and radiographic data were compared based on patients' demographic parameters (age, gender, BMI, and smoking) and the number of osteotomies.

### 2.9. Clinical Functional Outcome Measures

The clinical preoperative evaluation included a complete clinical history of the patients, their main characteristics (age, gender, BMI, dominant side, smoking, occupation, and anesthesia ASA class), and physical examination of the foot for preoperative planning, as well as the percutaneous procedures to perform (number of metatarsals to treat). To evaluate clinical outcomes at the preoperative period and last follow-up (FU), the following and most used questionnaires for forefoot assessment were used according to our study protocol:

- The 100-point AOFAS hallux metatarsophalangeal-interphalangeal scale [35, 36] was the only questionnaire used to assess clinical outcomes at the different FU points (preoperatively, 3-, 6-, and 12-month FU), and the difference of median values (Δ) between preoperative and the last evaluation was calculated;
- The Foot Functional Index (17-FFI) [35, 37] to measure the persistence of pain, disability, and restriction of activity with 17 number rating scales from 0 to 10;
- The Manchester-Oxford Foot Questionnaire (MOXFQ) [35] to establish how frequent the restrictions in specific situations were, including 16 questions divided into three basic domains: pain (five), walking/standing (seven), and social interaction (four);
- The Short Form 36 (SF-36) to identify the overall health reported by the subjects;
- The Visual Analog Scale (VAS) to quantify patient satisfaction with a score from 0 to 10.

Finally, during the last clinical check-up, patients were asked about their ability to work and possible modification of their daily activities. Additionally, any complications were recorded.

### 2.10. Radiographic Outcome Measures

**Figure 5:**
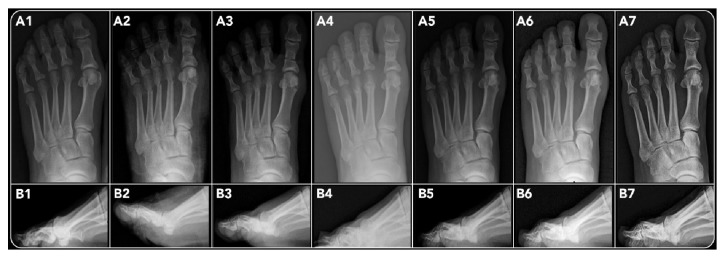
*(Case 1):* a 47-year-old female patient having undergone DMMO of the M2, M3, and M4 in addition to Reverdin-Isham and Akin percutaneous osteotomies for HV correction of her left foot. Radiographic images of anteroposterior (A) and lateral views (B): at preoperative period (1), immediate postoperative period (2), 1-month follow-up (3), 3-month follow-up (4), 6-month follow-up (5), 12-month follow-up (6), and 52 months after surgery (7), showing bone callus consolidation and its remodeling, maintaining the shortening and elevation of the head of the MBs treated.

**Figure 6:**
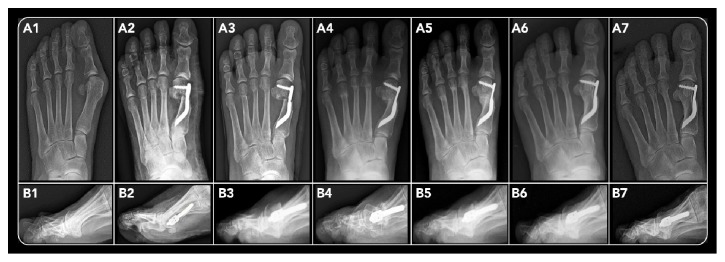
*(Case 2):* a 58-year-old female patient having undergone DMMO of the M2, M3, and M4 in association with Endolog technique for HV correction of her left foot, radiographic images of anteroposterior (A) and lateral views (B): at preoperative period (1), immediate postoperative period (2), 1-month follow-up (3), 3-month follow-up (4), 6-month follow-up (5), 12-month follow-up (6), and 48 months after surgery (7), showing bone callus consolidation and its remodeling, maintaining the shortening and elevation of the head of the MBs treated.

Routine standing anteroposterior and lateral X-ray views were obtained before surgery, at discharge, at one-month after surgery, and at different FUs (3-, 6-, 12-month, and last FU), according to our study protocol (Figures 5 and 6), respecting the Maestro method (weight-bearing, 15°-30° inclination of the X-ray beam, 1 m distance from the foot to the X-ray source). They were analyzed at our institution in a standardized manner using electronically computer-assisted Maestro measurements for weight-bearing radiographs provided by our MedStation program (the X-ray database of our hospital). This software allows the retrieval of electronically computer-assisted measurements from weight-bearing radiographs to minimize investigator bias.

Our sample was classified radiographically according to Maestro and Besse criteria [9], adding to this classification one more group to include those feet that did not reflect any morphotype as defined by Maestro parameters (Table 1). Hence, our cohort was divided into the following:

1. *Harmonious Morphotype, “normal forefoot,” *characterized by the SM4 line starting from the center of the lateral sesamoid bone and passing through the middle third of the 4th metatarsal (M4) head, perpendicular to the sagittal foot axis, and a geometrical progression of 2 of the lesser metatarsals;
2. *Nonharmonious Morphotype-1,* characterized by the SM4 line passing through the middle third of the M4 head, but the geometric progression of the lesser metatarsals is altered; M2 and M3 are too long;
3. *Nonharmonious Morphotype-2 is* the “*M4M5 hypoplasia*” morphotype, in which the SM4 line is disjointed and the perpendicular line to the M2 axis from the center of the sesamoid bone is distal to the middle third of the M4 head; the perpendicular line from the fifth metatarsal tip to the sagittal M2 axis is proximal to the distal pole of the lateral sesamoid bone (0.5–2 cm in severe hypoplasia);
4. *Nonharmonious Morphotype-3, *“M1 long,” characterized by a geometrical progression of 2 of the lesser metatarsal, which is generally correct; however, the SM4 line is displaced distally because the lateral sesamoid migrates distally with the M1 head.
5. *Unclassified Nonharmonious Morphotype: *this last group (our addition) includes all patients who do not belong to any of the forefoot morphotypes described by Maestro.

The radiographic evaluations included the Maestro criteria index [9] using the preoperative and the last FU anteroposterior standing X-rays. The relative length of each metatarsal was determined by drawing a line perpendicular to the axis of the foot and then measuring the distances (in millimeters) from each metatarsal head to this line (Figure 1), while also taking into account the relationship between the length of M1 and the length of the remaining MBs.

In anteroposterior and lateral standing view radiographs, callus formation and the absence of radiolucent lines were checked to determine bone union at the different FUs. Complete osteotomy healing time was then analyzed in relation to the main patient parameters and procedure variables, according to the study protocol (age, sex, BMI, smoking status, and number of MBs treated), in order to verify possible statistically significant correlations.

### 2.11. Statistical Analysis

Statistical analyses were performed by an independent statistician from the Department of Statistics at our University. The data is presented as the mean (plus standard deviation) or median (range) for continuous variables and as numbers for categorical measures. For the statistical evaluation of the clinical and radiological scores obtained with the various scales and the parameters of the Maestro formula before surgery and at last FU, we used Student's t-test. The Wilcoxon test was used to analyze the relationship between the variations of the healing time and the following variables: age, BMI, smoking status, and the number of metatarsals on which we performed osteotomies. Statistical significance was considered for* p* < 0.05.

## 3. Results

### 3.1. Patient Data

During a five-year period, 131 Caucasian patients (131 feet) with diagnosis of persistent central metatarsalgia were treated by DMMO in a single foot at our institution. We could not evaluate 38 patients (38 feet) as 12 refused to participate in FU assessment (one was unsatisfied with the clinical results and refused to come back for evaluation, and the remaining 11 were pleased with their results but were unable to come for evaluation); 6 did not complete clinical and/or radiographic assessment of the different FU points according to our postoperative control, while at the time of the last FU evaluation, 7 were located in a home for the elderly, 2 were dead, and a FU address could not be retrieved for 11 people. Hence, 93 patients (93 feet) completed different FUs until the last one according to the study protocol (Table 2). There were 14 men (15.1%) and 79 women (84.9%). At the time of surgery, the mean age was 62.4 ± 13.4 (range 31 to 87) years. The average FU period was 58.7 ± 12.7 (range 36 to 96) months, and most of the patients (53; 56.9%) were operated on between 60 and 79 years of age. In 43 cases (46.2%), the dominant limb was affected, while the nondominant limb was affected in 50 cases (53.8%). Regarding risk factors, 16 patients were obese (17.2%), 21 (22.6%) were active smokers, and 22 (23.7%) had comorbidities (hypertension, BPCO, and vascular disease). Hence, according to the ASA (American Society of Anesthesiologists) classification for globally estimated surgical risk, there were 56 ASA 1 patients (60.2%), 26 ASA 2 patients (28%), and 11 ASA 3 patients (11.8%).

During the 93 single foot operations, 198 DMMOs were performed as follows: osteotomies were localized only on the M2 in 23 feet (24.7%), on M2 and M3 in 35 (37.6%), and finally on three metatarsals (M2-M3-M4) in 35 (37.6%) (Table 3). In 86 (92.5%) of the 93 feet, associated procedures were performed tailored to patient's clinical presentation. Reverdin-Isham percutaneous osteotomy was performed in 29 feet for the correction of mild-moderate HV deformities and Endolog technique in 52 feet for moderate-severe ones, followed by percutaneous Akin osteotomy and percutaneous lateral soft-tissue release in some cases, according to our protocol. Further, we carried out flexor and extensor tenotomies in 31 feet for the correction of claw toe flexible deformities and associated osteotomies of the proximal phalange in 16 feet for the correction of fixed ones.

### 3.2. Clinical Functional Outcomes

**Figure 7:**
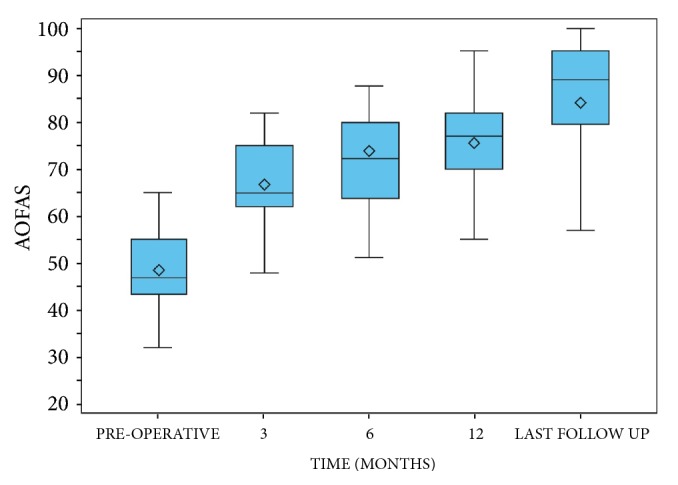
Graph of the statistical analysis of AOFAS scores at preoperative period, 3-6-12 months after surgery, and mean last follow-up of 58.7 months.

**Figure 8:**
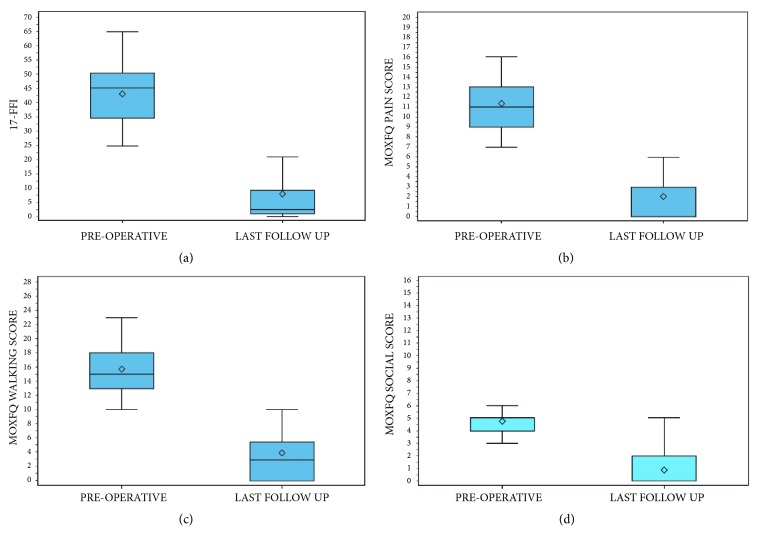
Graphs of the statistical analysis of 17-FFI (a), MOXFQ-PAIN (b), MOXFQ-WALKING (c), and MOXFQ-SOCIAL (d) scores (*p* < 0.0001), shown for each of the results at preoperative period and at mean last follow-up of 58.7 months.

At the preoperative evaluation, the mean AOFAS score of the patients was 48.6 ± 7.3 (range 22 to 65) points. Limitation in daily and recreational activities was present in 49 cases (52.7%). At different FU points, the mean AOFAS score was 66.6 ± 8.4 (range 42 to 82) points, 72.3 ± 10.6 (range 42 to 87) points, and 75.6 ± 12.1 (range 35 to 95) points at 3-, 6-, and 12-month FU, respectively, while it was 84.1 ± 14.4 (range 35 to 100) points at last FU. Hence, in our cohort, the AOFAS score improved significantly after surgery with respect to the preoperative value (*p* < 0.0001), reporting good and excellent results in 79 (84.9%) feet (Figure 7). The mean preoperative 17-FFI was 43.2 ± 9.7 (range 24.7 to 64.7) points, while the average at last FU was 7.8 ± 12.2 (range 0 to 54.6) points. Hence, also the 17-FFI improved significantly after surgery with respect to the preoperative value (*p* < 0.0001). The mean MOXFQ-Pain, -Walking, and -Social preoperative scores were 11.4 ± 2.4 (range 7 to 16) points, 15.8 ± 3.7 (range 10 to 23) points, and 4.8 ± 1.4 (range 0 to 8) points, respectively, while their mean values at last FU were 2.1 ± 3.1 (range 0 to 16) points, 3.9 ± 4.5 (range 0 to 24) points, and 0.8 ± 1.6 (range 0 to 8) points, respectively (Figure 8). The two components of the SF-36 (ISF and ISM) had, respectively, an average of 49.5 ± 8.6 (range 23.2 to 63.9) points and 49.5 ± 8.6 (range 23.2 to 63.9) points preoperatively, while being 49.5 ± 8.6 (range 23.2 to 63.9) points and 50.7 ± 7.5 (range 11.4 to 63.6) points at last FU, respectively. The mean VAS score was 2.4 ± 1.7 (range, 0 to 6) points at last FU, with respect to 5.1 points at the preoperative period. Among the patients operated on, at last FU, 49 (52.7%) kept their job after surgery and 2 (2.1%) had to change. The other patients were retired (23.7%) or homemakers (22.6%). In addition, 73 (78.5%) patients did not change their shoes. There was a reduction of patients with hyperkeratosis (from 77.4% to 9.7%) and MTP instability (Lachman test positive from 41.9% to 19.4%), as well as clinical signs of dorsal subluxation (from 15.1% to 6.5%) of these patients.

### 3.3. Radiographic Outcomes

In our sample, all radiographic parameters of the Maestro criteria were significantly different at the last FU compared to the preoperative period (*p *< 0.001). The forefoot* Harmonious Morphotype*, presented in 7 cases (7.5%) in the preoperative period, was identified only in 3 cases (3.2%) at last FU. A* Nonharmonious Morphotype-1* was present in 30 cases (32.3%) preoperatively with respect to 19 cases (20.4%) at last FU. A* Nonharmonious Morphotype-2* was present in 40 cases (43%) preoperatively with respect to 17 cases (18.3%) at last FU, and an* Unclassified Morphotype* was present in 16 cases (17.2%) with respect to 54 cases at last FU (58.1%). No cases of forefoot* Nonharmonious Morphotype-3* were found (Table 1).

Signs of bone callus formation were observed in 71 out of 93 feet (76.3%) at 3 months FU, while the other patients (23.7%) presented signs of osteotomy consolidation at 6 months postoperatively. Hence, all osteotomies were healed at 6 months after surgery (Figures 5(A5)-(B5) and 6(A5)-(B5)). The analysis of the possible variation of healing time based on patient parameters considered (age, sex, BMI, smoking, and number of metatarsals operated on) did not show statistically significant correlation: age (*p *> 0.1621), gender (*p *> 0.5923), BMI (*p* > 0.3234), smoking habits (*p *> 0.9638), and number of metatarsals operated on (*p *> 0.3571).

### 3.4. Complications

Short-term complications were present in 31 cases (33.3%): swelling (27 patients, 29%) and paresthesia (6, 6.4%), all resolved within 3 months from surgery. In addition, there were 23 cases (24.7%) of delayed union, completely healed at 6-month FU. Because of portal burns during operation, 3 patients (3.2%) presented delayed wound healing, which healed completely in four weeks and did not require subsequent surgery. Long-term complications were recorded in 12 cases (12.9%): 9 cases (9.7%) of persistent stiffness (ROM: < 30°) and 3 cases (3.2%) of transfer metatarsalgia on the M4. The latter patients were subjected to additional DMMO of the symptomatic MB. There were no infections, nonunion or malunion of the osteotomy, floating-toe deformity, soft tissue complications, residual instability or subluxation, or evidence of avascular necrosis of the metatarsal head.

## 4. Discussion

In the last fifteen years, DMMO became popular in Europe by the Spanish surgeon De Prado first and then by the GRECMIP (Groupe de Recherche et d'Enseignement en Chirurgie Mini-Invasive du Pied) as an alternative surgical technique to the traditional Weil osteotomy. Because of the perceived potential advantages of a dynamic correction offered by DMMO, and the stiffness and floating toe caused by Weil osteotomy [19], the percutaneous procedure now tends to be preferred over open osteotomies [10, 23]. However, the optimal treatment of metatarsalgia and the restoration of an ideal forefoot morphotype remain controversial [2, 3].

The purposes of this prospective study were first to specifically evaluate the safety and effectiveness of DMMO in treating persistent central primary metatarsalgia, identifying possible contraindications. The second was to verify the potential of DMMO in restoring a harmonious foot morphotype according to Maestro criteria and if these radiographic criteria are correlated with clinical outcomes, maintaining a predictive value for these criteria in the preoperative planning for this technique.

In our cohort, clinical outcomes improved significantly in each clinical score after surgery with respect to preoperative values, and the clinical results at the last FU were satisfactory (Figures 7 and 8). The AOFAS and MOXFQ scores over time showed a statistically significant improvement (*p* < 0.0001). The 17-FFI scores, which evaluate the clinical-functional appearance of the foot, showed excellent results with a statistically significant difference between pre- and postoperative periods (*p* < 0.0001). A significant reduction of pain (VAS scale: 2.4 vs 5.1 points) and formation of plantar callosities was obtained, associated with a considerable improvement in the quality of daily life in our patients as shown by SF36 (*p* < 0.0001). Further, osteotomy consolidation was present in 76.3% of feet after 3 months from surgical operation and in 100% at 6-month FU. The incidence of recurrence or transfer lesions was negligible. Henry [17] found similar healing times for DMMO, confirming that this technique requires longer healing time than the Weil osteotomy but with better results. Further, our results showed no variation of healing time based on age, sex, BMI, smoking, and number of metatarsals treated.

Jardé [18] affirmed that the patients in whom postoperative alignment of the metatarsal heads after Weil osteotomies had most closely met the Maestro criteria had better results than those in whom the match was less exact [9, 14]. However, in our patient group, reconstruction of an ideal curve of Maestro was obtained only in three of 93 of feet (3.2%), despite precise preoperative planning (Table 1). Hence, we found no correlation between having a harmonious and mathematically correct distal metatarsal parabola and clinical outcomes. In fact, our study showed that although the restoration of the ideal harmonious architectural Maestro curve of the forefoot was not achieved by DMMO, the procedure guaranteed balanced redistribution of the plantar pressure forces and relief of metatarsalgia not only in the immediate postoperative period, but also at medium-long term FU.

In our series, 81 feet (87%) were operated on concurrently for associated HV, in which instability of the first ray is implicated and can be a predisposing or exacerbating cofactor for metatarsalgia. This hypermobility is usually radiographically and clinically assessed in both sagittal and coronal planes, in the latter, in dorsal and also in dorsomedial directions, as recently proposed [38]. Similarly, a more complete approach to preoperative planning for metatarsalgia treatment, such as the research for new criteria and morphotypes in the coronal plane, should be promoted, as in a previous study of the preoperative planning of the Weil osteotomy [27]. In fact, Bevernage concluded that radiographic preoperative planning for metatarsalgia treatment only in the anteroposterior plane is clearly a simplification of a complex pathology because this pathology requires a three-dimensional correction [27]. The ideal preoperative planning for the more recent DMMO should take into account the potential proximal shift of the osteotomized metatarsal heads in both sagittal and coronal planes. However, this assessment is difficult to carry out as it should foresee the metatarsal head displacement not only at the operating table but also after load resumption by walking in the postoperative period, identifying new radiological and biomechanical criteria for the ideal foot morphotype. From our results, it is questionable if Maestro parameters can adequately represent this, maintaining their predictive value. After DMMO, the metatarsal heads consolidate into a more proximal position due to mechanical loading allowed after surgery by weight-bearing. This is in contrast to the Weil procedure, where the osteotomies, synthesized by screws, remain fixed after surgery, reflecting the ideal position calculated by the preoperative planning. For these reasons, it is the opinion of the authors that there is a need for revised radiological criteria that correlate radiological metatarsal alignment with clinical outcomes before and after this percutaneous technique.

Numerous complications related to DMMO have been reported [14, 19, 32, 39], but in our study, we had a low rate of short-term complications, such as transitory swelling, paresthesia, and skin burns, while delayed unions were resolved by 6-month FU. The main long-term complication was persistent stiffness (9.7%), and 3 cases (3.2%) of transfer metatarsalgia were resolved with a second percutaneous operation by M4 osteotomy. We had no cases of floating-toe deformity, residual instability or subluxation, infection, pseudarthrosis, avascular necrosis, or displacement of the metatarsal head, which are the most undesirable complications [14, 15, 22, 32]. We did not identify any risk factors that increased complications in our group. Redfern does not recommend DMMO in the presence of significant arthritis and stiffness of MTPJ because it is associated with increased risk of pseudarthrosis. However, this aspect was one of our exclusion criteria.

To the best of our knowledge, this is the first prospective, single-center study reporting clinical and radiographic outcomes of DMMO for the treatment of primary central metatarsalgia in a consecutive, single-surgeon patient series. The number of patients and the mean FU of almost 60 months were superior to previous published studies [25, 26, 30, 32]. For clinical evaluation of our sample, internationally validated scores were used, except for the AOFAS score, which, although it remains the most widespread health measurement in foot and ankle clinical practice, has been only partially validated [36]. For radiographic evaluation, the traditional method of Maestro [9] was used to perform X-ray foot images and to classify them both pre- and postoperatively, even to determine which MBs to shorten by DMMO during preoperative planning. The findings of this study should be interpreted within the context of its limitations. They are partially linked to the limited scientific literature on this subject and the poverty of cases of pure metatarsalgia. Most of the feet in our cohort included HV deformity, causing a potential bias of this study, and plantar pressure measurements were not performed.

## 5. Conclusion

First, Distal Metatarsal Metaphyseal Osteotomy (DMMO), often associated with percutaneous or open techniques for HV correction and percutaneous soft tissue and/or bone procedures in cases of lesser toe deformities, is a safe and effective minimally invasive method for the treatment of biomechanical central metatarsalgia. Further, age, gender, BMI, and smoking are not potential contraindications. Thus, DMMO can be considered a suitable alternative technique to traditional ones. Second, during preoperative planning, Maestro criteria were proved to be useful to calculate which MBs needed to be shortened to prevent transfer metatarsalgia and a significant clinical improvement of all scores assessed was noted at last follow-up. However, the ideal harmonious curve of the forefoot was restored by DMMO only in a few feet and a limited number of harmonious forefoot morphotypes were consequently found in the postoperative period. Hence, our data show that Maestro criteria do not have a predictive value in clinical outcomes of DMMO. Additional studies are required to find radiographic measurements more specific for the preoperative planning of this percutaneous technique.

## Figures and Tables

**Table 1:** Classification of forefoot morphotypes according to Maestro criteria, including the added unclassified ones.

**Forefoot ** **Morphotypes**	**Maestro Radiographic Parameters**	**Percentage usually in the ** **normal population**	**Study Cohort**	
			**Preoperative**	**Last Follow-up**
**Harmonious**	Harmonious Geometrical progression of 2 of the lesser M-bones with tolerance of 20% (±1 mm for M2M3 and M3M4, ± 2mm for M4M5)	31%	7.5% (7/93)	3.2% (3/93)

**Non-harmonious 1**	M2M3 increased with M3M4 and M4M5 normal; M2M3 decreased with M3M4 increased and M4M5 correct; M2M3 normal with M3M4 increased and M4M5 correct; M2M3 and M3M4 increased with M4M5 correct.	30%	32.3% (30/93)	20.4% (19/93)

**Non-harmonious 2**	M4M5 hypoplasia: M3M4 and M4M5 increased	37%	43% (40/93)	18.3% (17/93)

**Non-harmonious 3**	M1 > M2	2.4%	0% (0/93)	0% (0/93)

**Unclassified Non-harmonious**	Feet that did not reflect any Morphotype	-	17.2% (16/93)	58.1% (54/93)

**Table 2:** Patients' characteristics and their potential risk factors: mean (± standard deviation) and absolute (and relative) frequency (%).

**Demographic Parameters**	**Value**
**Gender**	
Males	14/93 (15.1)
Females	79/93 (84.9)
**Age (years)**	62.4 ± 13.4
30 - 39	7/93 (7.5)
40 - 49	11/93 (11.8)
50 - 59	17/93 (18.3)
60 - 69	27/93 (29)
70 - 79	26/93 (27.9)
≥ 80	5/93 (5.4)
**BMI (kg/m** ^**2**^ **)**	25.7 ± 4.3
>30	16/93 (17.2)
**Smoking**	
Yes	21/93 (22.6)
No	72/93 (77.4)
**Right feet**	
Dominant	78/93 (83.9)
**Left feet**	
Dominant	15/93 (16.1)
**Comorbidities**	
Hypertension	2/93 (2.2)
COPD	19/93 (20.4)
Vascular disease	1/93 (1.1)
**ASA**	
1	56/93 (60.2)
2	26/93 (28)
3	11/93 (11.8)

***Abbreviations***: ASA: *American Society of Anesthesiologists*; BMI: *Body Mass Index*; COPD: *Chronic Obstructive Pulmonary Disease*.

**Table 3:** Number of the metatarsal bones operated on according to the foot side and number of osteotomies performed by DMMO in our series.

**Site of osteotomy**	**Feet treated by DMMO**			**Number of DMMO Performed**
	***Right feet***	***Left feet***	*Total*	

**M2**	11 (24.4%)	12 (25%)	23 (24.7%)	23 (11.6%)
**M2-M3**	17 (37.8%)	18 (37.5%)	35 (37.6%)	70 (35.4%)
**M2-M3-M4**	17 (37.8%)	38 (37.5%)	35 (37.6%)	105 (53%)
*Total*	45 (48.9%)	48 (51.6%)	93 (100%)	198 (100%)

## Data Availability

The data used to support the findings of this study are available from the corresponding author upon request.
